# Coupling Multi Angle Light Scattering to Ion Exchange chromatography (IEX-MALS) for protein characterization

**DOI:** 10.1038/s41598-018-25246-6

**Published:** 2018-05-02

**Authors:** Hadar Amartely, Orly Avraham, Assaf Friedler, Oded Livnah, Mario Lebendiker

**Affiliations:** 1 Wolfson Centre for Applied Structural Biology, The Alexander Silberman Institute of Life Sciences, The Hebrew University of Jerusalem, Safra Campus, Givat Ram, Jerusalem, Israel; 2 Department of Biological Chemistry, The Alexander Silberman Institute of Life Sciences, The Hebrew University of Jerusalem, Safra Campus, Givat Ram, Jerusalem, Israel; 3 Institute of Chemistry, The Hebrew University of Jerusalem, Safra Campus, Givat Ram, Jerusalem, Israel

## Abstract

Multi-angle light scattering coupled with size exclusion chromatography (SEC-MALS) is a standard and common approach for characterizing protein mass, overall shape, aggregation, oligomerization, interactions and purity. The limited resolution of analytical SEC restricts in some instances the accurate analysis that can be accomplished by MALS. These include mixtures of protein populations with identical or very similar molecular masses, oligomers with poor separation and short peptides. Here we show that combining MALS with the higher resolution separation technique ion exchange (IEX-MALS) can allow precise analyses of samples that cannot be resolved by SEC-MALS. We conclude that IEX-MALS is a valuable and complementary method for protein characterization, especially for protein systems that could not be fully analyzed by SEC-MALS.

## Introduction

Light scattering (LS) based techniques are commonly used in the research and characterization of different molecules and particles^1,2^. In particular, multi-angle light scattering (MALS) is utilized for molar mass determination of macromolecules such as proteins according to Rayleigh theory, and dynamic light scattering (DLS) can be used for measuring the hydrodynamic radius of the protein^3^. Characterization of a mixed sample using MALS can be performed for several types of macromolecules such as polymers, though for protein characterization MALS should be utilized with a pure sample or coupled to a separation technique^4^. Combining MALS with analytical separation techniques allows molar mass calculation at any point in the elution chromatogram and characterization of each population in a mixed sample. Size exclusion chromatography (SEC) and field flow fractionation (FFF), which separate macromolecules by size, are the frequent separation techniques coupled in-line with MALS for biological macromolecules characterization^3–5^.

SEC is a liquid chromatography method that separates macromolecules of different sizes based on the partial exclusion of these molecules from the pores of the stationary phase^6^, and is widely used as an analytical tool for protein characterization and for molecular mass estimation^7^. However, mass estimation by SEC is often inaccurate, since the retention time of the macromolecule depends on its hydrodynamic radius and not only on its molecular mass. In addition, the interactions that could occur between the macromolecule and the stationary phase may affect the retention time relative to the theoretical expected value for the studied sample^4,7^. Coupling an analytical SEC column in-line with multi-angle light scattering, UV detector and refractometric detector (SEC-MALS) provides a widespread approach and a useful tool for accurate analysis of molar mass, oligomeric states and hydrodynamic radius of proteins in native solution, independent of the protein retention time analyzed by SEC^3,8,9^. SEC-MALS is also useful for studying and characterizing aggregations occurring in protein samples due to the high sensitivity of light scattering to high molecular weights species^4,10^. The process of protein aggregation is an essential topic of substantial research and can results from different factors such as protein misfolding, chemical environment and concentration^11,12^.

Despite its advantages and potential, SEC-MALS has several limitations: 1. The technique separates proteins only by size, so molecules with the same size cannot be separated and properly analyzed; 2. For a suitable oligomeric analysis, SEC-MALS should be used only for well-resolved peaks^4^. However, most of the analytical SEC columns have limited separation ability due to their short length and thus full separation of oligomeric forms can be difficult; 3. Aggregates have a very intense light scattering signal, thus the presence of even low amounts of aggregates in the protein peak can introduce a significant error into the calculated molar mass. 4. SEC-MALS usually requires extensive equilibration for achieving a clean baseline signal, since SEC columns often release particles from the stationary phase that interfere with the light scattering measurements (“column shedding”)^4^; 5. Separation in analytical SEC is highly influenced by the injection volume and this volume limitation does not exist in other chromatographic procedures. Therefore, relatively high protein concentrations may be required (mainly for small macromolecules) in order to obtain enough LS signal for MALS analysis.

Ion exchange chromatography (IEX) is another chromatographic method commonly used for protein separation and characterization^13^. The principle of IEX is based on protein separation according to their surface charge, leading to different ionic interactions with the support matrix^14,15^. Anion exchange (AIEX) and cation exchange (CIEX) matrixes bind negatively and positively charged variants respectively. The most common use of IEX chromatography is as an intermediate purification step for separating target proteins from aggregates, host cell proteins and other contaminants. IEX columns can separate between different oligomeric states of a protein^16^, protein isoforms^17^ and modified proteins such as glycoproteins^18,19^. Separation with an IEX can be achieved by a classical linear salt or pH gradient^19–21^. Unlike SEC, numerous parameters can be optimized in IEX chromatography in order to improve resolution, such as the gradient slope, salt composition and concentration, buffer pH, type of ligand, matrix, and commercial sources.

Here we took advantage of the high resolution and fine separation capabilities of IEX chromatography combined with MALS. We thus present an analytical IEX-MALS approach for protein characterization. Similarly to SEC-MALS, the addition of a light scattering analysis allows molar mass measurements for each individual peak in the elution profile. However, since the principle of separation is entirely different and the resolution is higher than SEC, analytical IEX-MALS can provide complementary information to SEC-MALS and can often resolve the SEC-MALS impairments described above.

## Results and Discussion

### IEX-MALS technique

**Figure 1:**
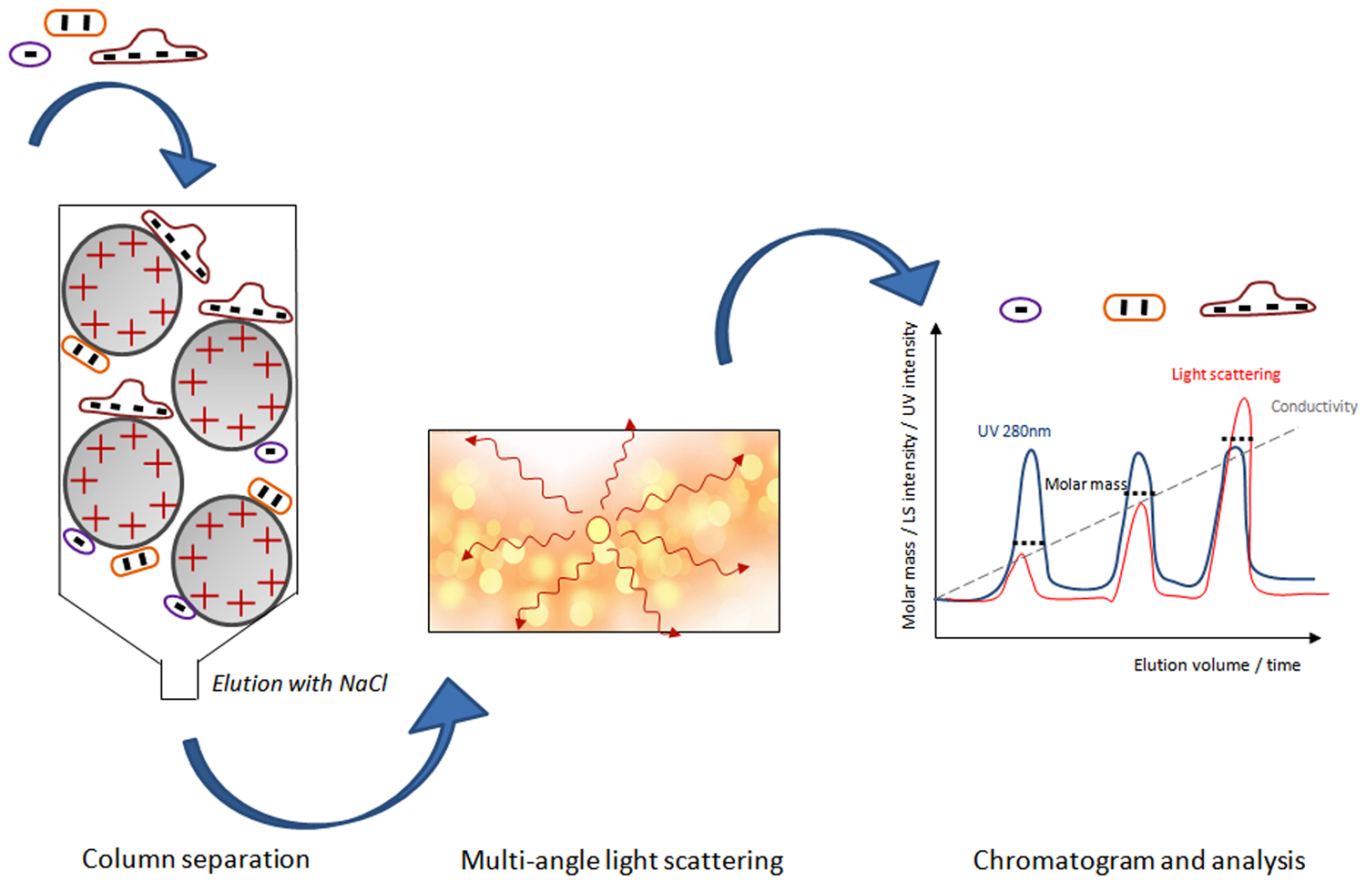
Schematic illustration of the IEX-MALS method. A proteins-mixed sample is injected into an IEX chromatography column in-line with a multi-angle light scattering detector. Proteins interact with the charged matrix and elute form the column with increased salt gradient according to the strength of binding: highly charged proteins bind stronger to the matrix and elute with higher salt concentration.

The principle of IEX-MALS is an in-line combination of an IEX chromatography (AIEX or CIEX) with the MALS system. The MALS used in this study is a miniDAWN TREOS (Wyatt Technology) with three angle LS detectors (see materials and method section). As illustrated in Fig. 1, proteins bind the stationary phase based on their overall charge, and the density and distribution of their surface charge. Elution of the bound proteins from the column is performed via salt gradient, such as NaCl. A weakly bound protein elutes from the matrix at low salt concentrations (low conductivity) and strongly bound protein elutes at higher salt concentrations (high conductivity). During elution from the column the molecules are introduced to the MALS system where light scattering at several angles is measured, together with the dynamic light scattering and the refractive index signals. This allows calculations of the molar masses and hydrodynamic radii for each peak eluted utilizing IEX chromatography.

### Development of IEX-MALS

**Figure 2:**
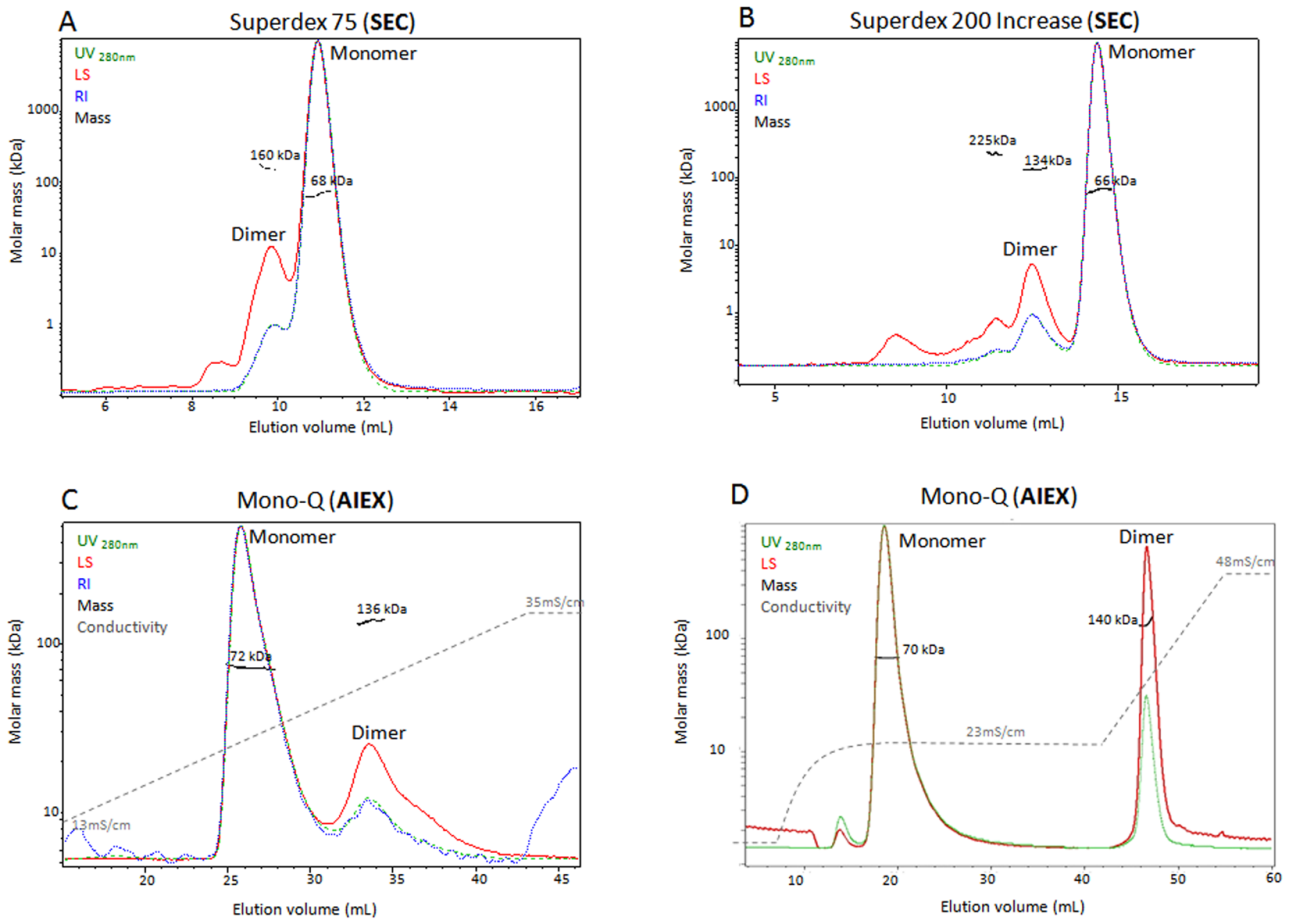
SEC-MALS and AIEX-MALS of BSA. BSA was separated and analyzed using Superdex 75 (**A**) and Superdex 200 increase (**B**) analytical SEC columns and an AIEX analytical column Mono-Q with different gradient programs (**C**,**D**) and consequently with MALS. The four chromatograms display the UV at 280 nm (green), light scattering at 90° angle (red), refractive index (blue) and conductivity (grey) curves together with the molar mass of each peak calculated by MALS (black).

To develop and assess the IEX-MALS technique vis-a-vis SEC-MALS, we used Bovine Serum Albumin (BSA) as our test protein comparing the MALS analysis obtained by both approaches. BSA is mainly present as a monomer (theoretical mass of 66.5 kDa) in solution, though it contains also a dimeric fraction and small amounts of higher oligomeric forms^22^. BSA was analyzed using SEC-MALS and AIEX-MALS with Superdex 75, Superdex 200 Increase and Mono-Q analytical columns, as described in the method section (Fig. 2). Both SEC columns indicate that BSA is mostly a monomer (66 ± 1 kDa according to Superdex 200 Increase and 68 ± 1 kDa according to Superdex 75), containing 15% oligomeric species according to Superdex 75 and 17% oligomeric species according to Superdex 200 Increase. Separation of these oligomers from the main monomeric peak is dictated by the specific sort of column. While in Superdex 75 the separation was limited (Fig. 2A), in Superdex 200 Increase a better separation was obtained (Fig. 2B). Good separation of the BSA monomer (72 ± 2 kDa) and dimer (136 ± 4 kDa) was also achieved on the AIEX column with a linear gradient of 75–350 mM NaCl (Fig. 2C). By changing the elution gradient program to a step of 175 mM NaCl followed by a linear gradient of 175–500 mM NaCl, the resolution was optimized and an excellent-separation of the monomeric (70 ± 4 kDa) and dimeric (140 ± 10 kDa) peaks was observed (Fig. 2D). Both AIEX experiments revealed that BSA contains 19% oligomeric species, in agreement with the SEC-MALS analysis. As opposed to SEC columns, in the AIEX method the oligomeric species elute after the monomer at higher conductivity due to stronger interactions with the matrix. In addition to gradient slope optimization, other parameters can be varied to increase resolution in IEX. These include pH gradient, type of salt, type of buffer and column matrix. These features allow better separation between oligomers and accurate molar mass calculations of the separated protein peaks. The change in salt concentration during the IEX-MALS experiment leads to a change in the solution refractive index, which results with a small change in the refractive index increment (dn/dc) value. All molar masses were calculated using the corrected dn/dc values for each peak due to the specific conductivity in the elution chromatogram, using equation 1 (see materials and methods). The refractive index (RI) signal, which is required for certain types of analysis such as conjugated proteins or unknown proteins, changes dramatically during the salt gradient in IEX-MALS experiments (see Supplementary Fig. S1). Therefore, a relatively high amount of solute is required for MALS analysis that utilizes RI. In addition, if the salt gradient is not linear, a buffer subtraction is also required for the IEX-MALS analysis. The conditions used in IEX experiments allow elution of proteins at relatively low salt concentrations, lower than 0.5 Molar. However, for a protein that elutes at high salt concentrations that are far from its native conditions, MALS analysis would not represent the native conditions of the protein.

### Separation of oligomers using IEX-MALS

**Figure 3:**
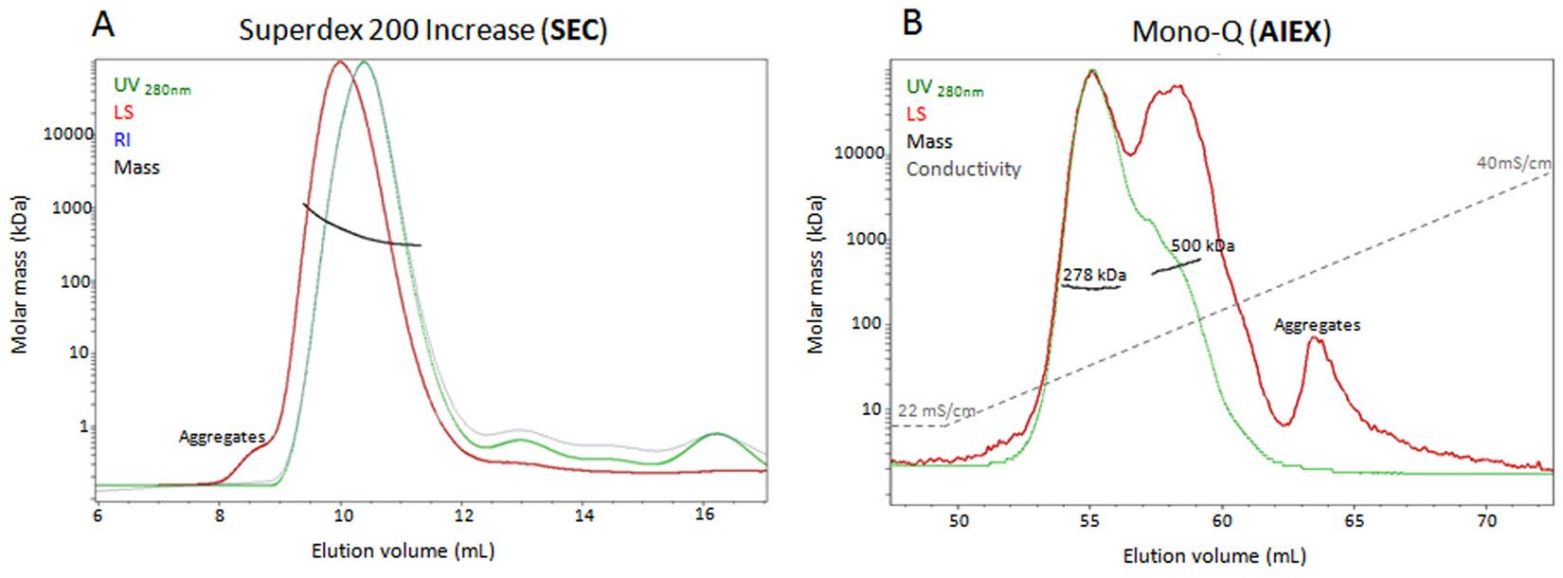
SEC-MALS and AIEX-MALS of fibronectin. Fibronectin was separated and analyzed using a Superdex 200 increase analytical SEC column (**A**) and an AIEX analytical column Mono-Q (**B**) and consequently by MALS. Chromatograms display the UV at 280 nm (green), light scattering at 90° angle (red), refractive index (blue) and conductivity (grey) curves together with the molar mass of the peaks determined by MALS (black).

Oligomeric species that exist in a protein sample often elute from an analytical SEC column with a poor separation profile leading to limited analysis by SEC-MALS and without definitive and clear conclusions. A good example for this limitation is the extracellular matrix protein fibronectin which regulates cell attachment, migration, differentiation, proliferation and survival^23^. Fibronectin is active as a dimer but is also present as a monomer and contains higher oligomeric states as well. SEC-MALS experiments of fibronectin display a poor separation profile of the oligomeric species using the Superdex 200 Increase column (Fig. 3A). SDS-PAGE gel of fibronectin reveals a highly pure protein sample, indicating that all eluted species are oligomeric states of fibronectin (Supplementary Fig. S2). All oligomers elute as one asymmetric peak demonstrating its heterogeneity. SEC-MALS analysis of molar mass also revealed heterogeneity within the peak, indicating that all forms elute at a similar retention time with no clear separation. An improved resolution was obtained using an AIEX column. AIEX-MALS experiments clearly separated the fibronectin monomers (with theoretical mass of 263 kDa and calculated mass of 278 ± 8 kDa) from the higher molecular species (calculated average mass of 500 kDa) and also from the aggregated fraction (Fig. 3B). As in the BSA example, monomers elute before higher oligomers and aggregates at lower conductivity values, resulting in a significantly improved molar mass analysis.

**Figure 4:**
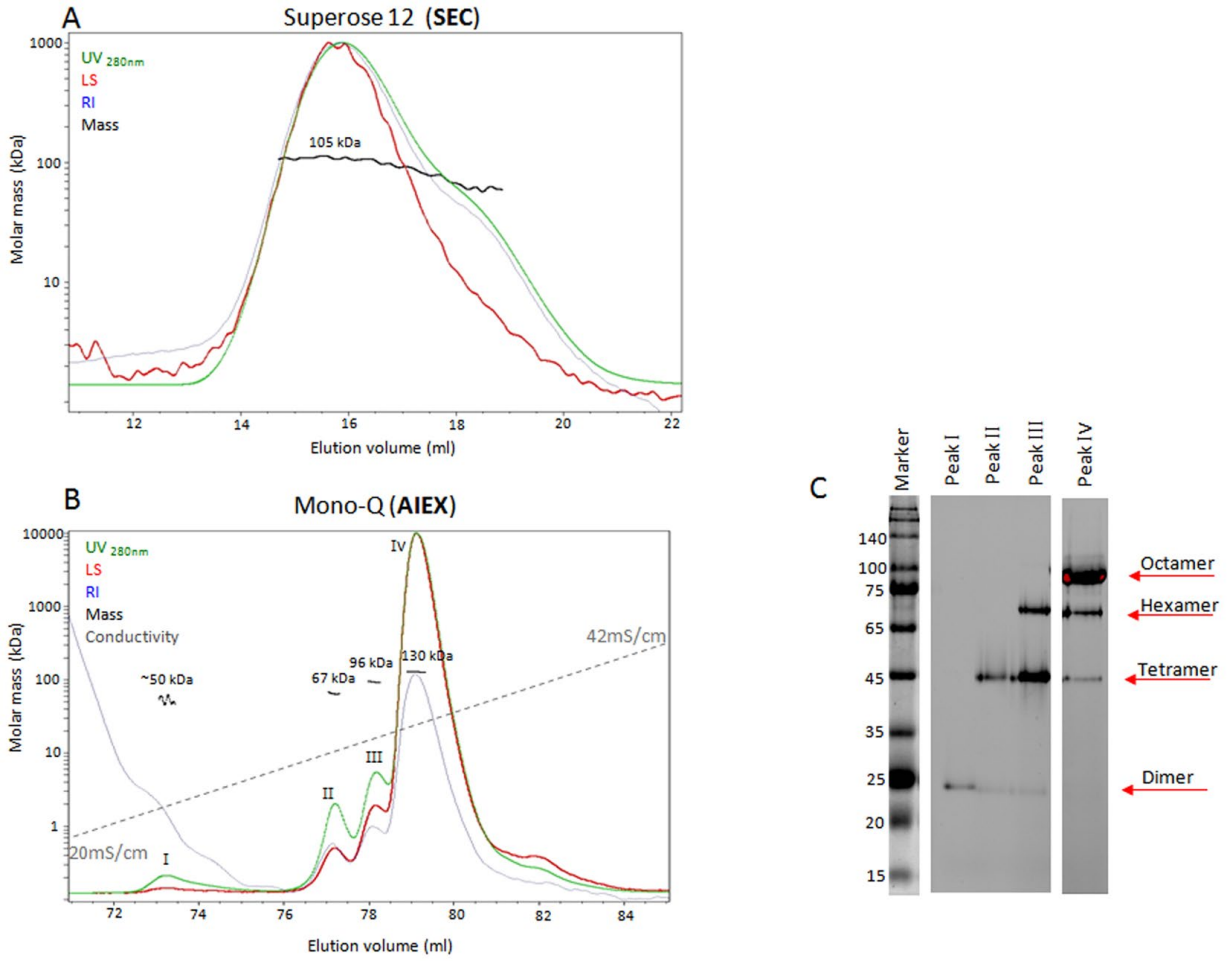
SEC-MALS and AIEX-MALS of the hoefavidin variant. The protein was separated and analyzed using a Superose 12 analytical SEC column (**A**) and an AIEX analytical column Mono-Q (**B**) in-line with MALS. Chromatograms display the UV at 280 nm (green), light scattering at 90° angle (red), refractive index (blue) and conductivity (grey) curves together with the molar mass of the peaks determined by MALS (black). (**C**) Gel image of native PAGE analysis of fractions from each peak in the AIEX-MALS experiment (see Supplementary Fig. S4 for full gel image).

An additional example of oligomeric separation accomplished by IEX-MALS is provided by a mutant variant of the hoefavidin protein, a member of the avidins family with a monomeric mass of 15.5 kDa, that exists mainly as a dimer and octamer^24^. The protein was analyzed using SEC-MALS and AIEX-MALS with Superose 12 and Mono-Q analytical columns (Fig. 4). SEC-MALS results show a wide peak followed by a non-separated shoulder (Fig. 4A). The calculated molar mass of the main peak was 105 ± 6 kDa and the molar mass of the shoulder could not be accurately analyzed due to poor separation from the higher oligomers. Analysis with AIEX-MALS shows an entirely different profile. In this context, hoefavidin was eluted from the Mono-Q column in four peaks, corresponding to different oligomeric states: dimer (50 ± 20 kDa), tetramer (67 ± 2 kDa), hexamer (96 ± 2 kDa) and octamer (130 ± 4 kDa) (Fig. 4B). These oligomeric forms were confirmed by molar mass calculations using MALS analysis and also by a native gel analysis using fluorescent biotin (Fig. 4C). Most of the analytical SEC columns that are commonly used for protein SEC-MALS measurements have a limited resolution due to their short length. When high-order oligomers or aggregates are not fully separated from the protein peak, the molecular mass determination of lower oligomers will be inaccurate. The significantly improved separation obtained with IEX-MALS in the above three examples (BSA, fibronectin and hoefavidin mutant) together with the fact that lower oligomers elute before higher oligomers facilitate a more accurate analysis by MALS.

### Using IEX-MALS to distinguish between proteins with a similar molecular mass

**Figure 5:**
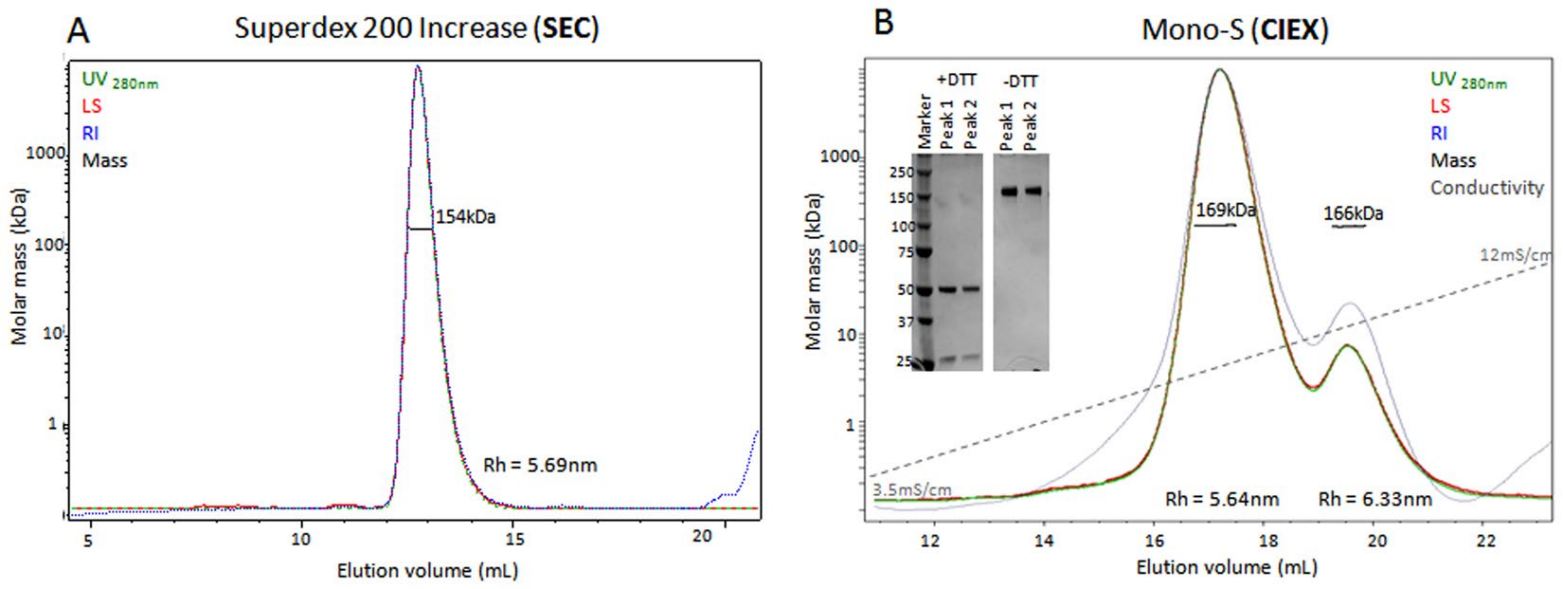
SEC-MALS and CIEX-MALS of Adalimumab. (**A**) SEC-MALS results of Adalimumab. (**B**) CIEX-MALS results of Adalimumab and a gel image of SDS-PAGE analysis of the eluted peaks in reducing (+DTT) and non-reducing (−DTT) conditions (see Supplementary Fig. S4 for full gel image). The chromatograms display the UV at 280 nm (green), light scattering at 90° angle (red), refractive index (blue) and conductivity (grey) curves together with the molar mass of each peak determined by MALS (black).

Proteins with highly similar sizes cannot be separated by SEC, but if they have different pI values they can be separated by IEX chromatography. Such an example is antibodies. Antibodies appear as two peaks in IEX chromatography corresponding to variants of the acidic and basic species^25^. Adalimumab (known commercially as Humira) is a therapeutic antibody used for treatment in several autoimmune diseases^26^. Adalimumab appears in CIEX as two peaks of the acidic and basic variants^27^. Adalimumab was analyzed using SEC-MALS and CIEX-MALS, with Superdex 200 Increase and Mono-S analytical columns (Fig. 5). The results via SEC-MALS show that Adalimumab exists as one homogeneous peak, with a mass of 154 ± 1 kDa and hydrodynamic radius of 5.68 ± 0.02 nm (Fig. 5A). Unlike SEC, the results of CIEX-MALS demonstrate clearly that Adalimumab contains two species with highly similar mass (169 ± 1 kDa and 166 ± 2 kDa for acidic and basic species respectively) but with different charge characteristics (Fig. 5B). Both peaks correspond to the same antibody molecule according to SDS-PAGE (Fig. 5B). The difference in the surface charge of the antibody variants can be due to different modifications, such as glycosylation, leading to conformational changes between them^25^. Our CIEX-MALS results show that the two variants exhibit different hydrodynamic radii (5.64 ± 0.02 nm and 6.33 ± 0.05 nm for the acidic and basic species respectively) which support the assumption of structural differences between the two variants. The viscosity of the solution changes with the salt gradient and this can introduce small errors in the calculated hydrodynamic radii. Therefore solvent parameters should be corrected and fit the salt concentration of the eluted protein to avoid these errors. The two variants of Adalimumab eluted at very similar conductivity, around 85 mM NaCl. Molar masses and hydrodynamic radii were calculated using the corrected parameters of the solvent for this conductivity value (see materials and methods). The antibody example emphasizes the fact that different proteins or populations that share a similar size cannot be separated by SEC but can be separated and analyzed successfully using IEX-MALS. When many variants of a protein exist in a sample, such as several glycosylation patterns that lead to different binding affinities of the protein to the IEX resin, separation between all populations can be difficult. An example is the Hen egg protein ovalbumin^28^ (43 kDa) that exists mostly as a monomer (as shown by SEC-MALS and SDS-PADE) but has many variants that bind differently to the Mono-Q column (Supplementary Fig. S3). Even with a specific and mild gradient program, separation in AIEX is limited therefore calculated molar masses are inaccurate. For such samples, and for samples with insufficient separation, a meticulously planned gradient or a stepwise program should be applied for separating one species from another. It should be noted that using a mild gradient increases the resolution of the chromatographic separation but usually results with lower concentration of the eluted protein, due to slow elution of the protein from the column, therefore higher concentrations of injected samples are required for precise analysis by MALS.

### Analysis of short peptides by IEX-MALS

**Figure 6:**
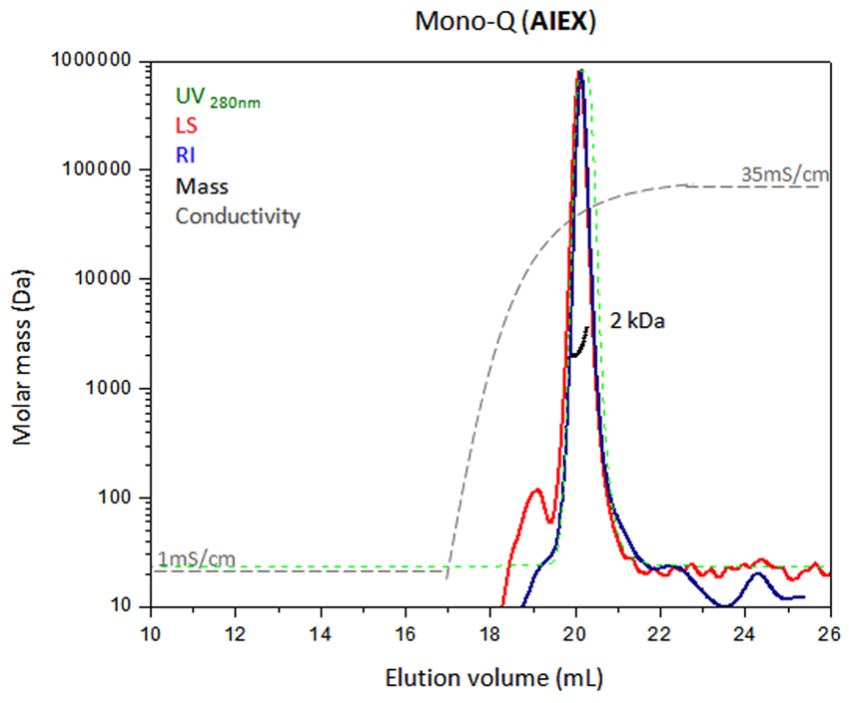
AIEX-MALS of a peptide. A tested peptide was analyzed using a Mono-Q analytical column in-line with MALS. The chromatogram displays the UV at 280 nm (green), light scattering at 90° angle (red), refractive index (blue) and conductivity (grey) curves together with the molar mass of the peak determined by MALS (black).

Although the MALS can analyze small macromolecules (~200 Dalton for the miniDAWN TREOS instrument), the SEC column has a limited separation range. Small macromolecules or peptides elute near the total volume of commercial and standard SEC columns, close to salts and buffer ingredients that are present in the analyzed sample. Therefore SEC-MALS analysis of such molecules can be challenging. In addition, since the intensity of light scattering depends on the molar mass and the concentration of the macromolecule^8^, a highly-concentrated peptide sample should be injected to obtain a minimal LS signal required for SEC-MALS analysis. Peptides can bind the IEX matrix due to their charge. Unlike SEC, in IEX there is no volume limitation and a sufficient volume of the analyzed sample is injected to the column and elutes highly concentrated, increasing the LS intensity. Moreover, IEX columns exhibit less “column shedding” effect compared to SEC^4^ which allows a shorter equilibration time to obtain a clean baseline LS signal. We analyzed a peptide (described in the Methods section) using AIEX-MALS. Figure 6 shows the elution profile of the peptide with the molar mass of 2.0 ± 0.6 kDa determined by MALS, consistent with the theoretical mass of 2,026 Da. The unlimited sample loading volume of IEX columns is an extremely important advantage of IEX-MALS over SEC-MALS for small macromolecules such as peptides but also for diluted samples and proteins that tend to aggregate upon concentration.

## Conclusions

IEX-MALS is a high-resolution method combining excellent separation of proteins with accurate determination of their molar mass. It can therefore be used not only for analysis of pure protein samples but also for heterogeneous samples. The different principle of separation used in IEX-MALS provides an additional and critical level of protein characterization and can overcome the SEC-MALS limitations described above (summarized in Table 1), in particular for protein samples that are not suitable for a typical SEC-MALS experiment. These make IEX-MALS a valuable method for protein characterization and for quality assessment, especially in cases where SEC-MALS analysis is insufficient or unsuccessful.

**Table 1:** Advantages and limitations of SEC-MALS vs. IEX-MALS.

Method parameters	SEC-MALS	IEX-MALS
Principle of separation	Hydrodynamic size	Charge
Parameters that increase selectivity and resolution	Restricted Different columns can be used with different fractionation range, resin particle size, matrix or different column length	Varied Different steps/gradient running programs, gradient slope, pH or salt gradient, type of salts, type of buffer, resin particle size, different matrixes, type of column (CIEX/AIEX) and column length
Injected volume	Limited	Unlimited^*^
Sample concentration	Concentrated sample	Diluted or concentrated sample
Sample buffer	As desired	Conditions that allow binding
Equilibration time	Long	Short
Flexibility of changing parameters during the run	Not flexible	Flexible
Conjugate analysis (for modified proteins) achieved by using RI signal	Easy to perform	More laborious RI signal changes during salt or pH gradients. Requires high sample concentration
Analysis of low masses	Not recommended	Possible
Analysis of mixtures of proteins with similar size	Not recommended	Recommended
Complexity of experiment	Easy and intuitive	Requires prior optimization or knowledge of conditions

*Injected volume is unlimited, although total amount of injected sample is restricted by the column capacity. For Mono Q HR 5/5 (GE) the loading capacity is ~25 mg protein^33^.

## Materials and Methods

### SEC-MALS experiments

A miniDAWN TREOS multi-angle light scattering detector, with three angles (43.6°, 90° and 136.4°) detectors and a 658.9 nm laser beam, (Wyatt Technology, Santa Barbara, CA) with a Wyatt QELS dynamic light scattering module for determination of hydrodynamic radius and an Optilab T-rEX refractometer (Wyatt Technology) were used in-line with several size exclusion chromatography analytical columns: Superdex 200 Increase 10/300 GL (GE, Life Science, Marlborough, MA), Superdex 75 10/300 GL (GE) and Superose 12 10/300 (GE). Experiments were performed using an AKTA explorer system with a UV-900 detector (GE), with the running protocols described for the following examples. All experiments were performed at room temperature (25 °C). Data collection and SEC-MALS analysis were performed with ASTRA 6.1 software (Wyatt Technology). The refractive index of the solvent was defined as 1.331 and the viscosity was defined as 0.8945 cP (common parameters for PBS buffer at 658.9 nm). dn/dc (refractive index increment) value for all samples was defined as 0.185 mL/g (a standard value for proteins).

### IEX-***MALS*** experiments

Same detectors (miniDAWN TREOS, Optilab T-rEX and AKTA explorer system with a UV-900 detector) were used in-line with an anion exchange chromatography analytical column Mono-Q HR 5/5 (GE) or with a cation exchange chromatography analytical column Mono-S HR 5/5 (GE). Experiments were performed with the running protocols described for the following examples. All experiments were performed at room temperature (25 °C). Data collection and IEX-MALS analysis were performed with ASTRA 6.1 software. The refractive index of the solvent and the viscosity were defined for PBS buffer. dn/dc values were corrected for each peak in the elution chromatogram of every sample based on the conductivity of the eluted peak. The refractive index of the solution slightly changes during NaCl gradient, therefore also dn/dc changes. Equation 1^29^ was used for the correction of dn/dc value for each eluted peak in IEX-MALS experiments:

1 $$dn/dc=\frac{n(protein)-n(solvent)}{\bar{v}}$$

The specific protein volume - $\bar{v}$ was determined as 0.73 mL/g, an average value for proteins^30^. Increase of 0.00085 in n (solvent), equals to increase of 85 mM NaCl, leading to decrease of 0.0011 in dn/dc^31^.

The change of salt during IEX-MALS experiments also leads to changes in the viscosity of the solution. For every 85 mM shift in NaCl concentration, a change of 0.008 cP in the viscosity is introduced^31^. For DLS measurements, the parameters of the solvent were changed to the parameters of the specific conductivity (described below).

RI baseline subtraction was performed using the ASTRA software method “*baseline subtraction*”.

It should be noted that large change in salt concentration during the elution gradient in IEX can affect the detectors normalization of the MALS and can introduce some errors mainly in the calculation of radius of gyration (R_g_). This can be encountered with a more than three angles MALS instrument.

### Design of ***an*** IEX experiment

Anion exchange (AIEX) or cation exchange (CIEX) chromatographies were selected based on the isoelectric point (pI) value of each tested protein. For proteins with pI higher than 7, a CIEX chromatography was used with a pH buffer lower than the pI. For proteins with pI lower than 7, an AIEX chromatography was used with a pH buffer higher than the pI. Selected pH was also determined due to the protein stability. First IEX experiment for each protein was a standard linear salt gradient (0–100% of elution buffer containing 0.5 M or 1 M NaCl). According to the specific conductivity value of the eluted protein peak, the gradient was optimized to increase separation of the desired protein species from the others or from contaminations.

### Proteins preparation and protocols for chromatographic runs

Commercial BSA (Sigma A1900) was dissolved in 20 mM Tris-HCl buffer pH = 8 with 50 mM NaCl and analyzed using SEC-MALS and AIEX-MALS. For SEC-MALS experiments, 100 µL of 5 mg/mL (0.5 mg) BSA were loaded on a Superdex 75 analytical column and on a Superdex 200 Increase analytical column with running buffer of 50 mM Tris-HCl pH = 7.5 and 150 mM NaCl. For AIEX-MALS experiments, 250 µL of 10 mg/mL (2.5 mg) or 400 µL of 7 mg/mL (2.8 mg) BSA were loaded on a Mono-Q analytical column using 20 mM Tris-HCl buffer pH = 8 and 50 mM NaCl. Elution was obtained by a 30 column volume (CV) gradient of 15–70% or by a 40CV of 35% step and 12CV gradient of 35–100%,using 20 mM Tris-HCl buffer pH = 8 and 500 mM NaCl as elution buffer.

Commercial ovalbumin (Sigma A-2512) was dissolved in 20 mM Tris-HCl buffer pH = 8 with 50 mM NaCl and analyzed using SEC-MALS and AIEX-MALS. For SEC-MALS experiments, 300 µL of 10 mg/mL (3 mg) ovalbumin were loaded on a Superdex 200 increase analytical column with PBS as running buffer. For AIEX-MALS experiments, 1 mL of 10 mg/mL (10 mg) ovalbumin were loaded on a Mono-Q analytical column using 20 mM Tris-HCl buffer pH = 8 and 50 mM NaCl. Elution was obtained by a 15CV gradient of 10–20% followed by a 7CV gradient of 20–50%,using 20 mM Tris-HCl buffer pH = 8 and 500 mM NaCl as elution buffer.

Commercial fibronectin (Sigma F1141) was analyzed with SEC-MALS and AIEX-MALS. For SEC-MALS experiment, 0.45 mg fibronectin were loaded on a Superdex 200 Increase analytical column with running buffer of 20 mM Tris-HCl pH = 8 and 100 mM NaCl. For AIEX-MALS experiment, 1 mg protein was diluted x20 with 20 mM Tris-HCl buffer pH = 8 and 20 mL of 0.05 mg/mL were loaded on a Mono-Q analytical column using 20 mM Tris-HCl buffer pH = 8 and 50 mM NaCl. Elution was obtained by a 30CV gradient of 15–45% using 20 mM Tris-HCl buffer pH = 8 and 1 M NaCl.

A mutant variant of hoefavidin^24^ was cloned into pET28a vector (Novagen) with Kanamycin resistance. Plasmid was transformed into BL21 (DE3) and grew at 37 °C. Induction was performed at OD_600nm_ of 0.6 with 0.4 mM isopropyl-β-D-1-thiogalactopyranoside (IPTG) for 5–6 hours at 30 °C. Bacteria were harvested and dissolved with a lysis buffer containing 50 mM Tris-HCl pH = 8, 0.3 M NaCl, 0.5 M Guanidinium chloride, 0.1 mM Glutathione disulfide (GSSG) and 1 mM Glutathione (GSH), followed by disruption with a microfluidizer LV1 (Microfluidics). Soluble fraction was separated by centrifugation (25 min, 4 °C, 16500 g) and purified with 2-iminobiotin resin (PIERCE) beads with the previously-described protocol^24^. 130 µL of 3.1 mg/mL (0.4 mg) protein was analyzed with SEC-MALS using a Superose 12 analytical column with 50 mM Tris-HCl pH = 8, 300 mM NaCl and 0.02% N_a_N_3_ as a running buffer. 2.3 mg protein was analyzed with AIEX-MALS using a Mono-Q analytical column. The protein was diluted in 40 mL of 20 mM Tris-HCl buffer pH = 8 and 50 mM NaCl before loading (final protein concentration 0.057 mg/ml). Elution was performed with a 20CV gradient of 25–100% using 20 mM Tris-HCl buffer pH = 8 and 500 mM NaCl.

Adalimumab (Humira, AbbVie 3799(53)) was analyzed using SEC-MALS and CIEX-MALS. For SEC-MALS experiment, 80 µL of 5 mg/mL (0.4 mg) Adalimumab were loaded on a Superdex 200 Increase 10/300 analytical column (GE) with running buffer of 20 mM sodium phosphate buffer pH = 6.2 and 100 mM NaCl. For CIEX-MALS experiment, 20 µL of 50 mg/mL (1 mg) protein were diluted x10 with 20 mM sodium phosphate buffer pH = 6.2 and loaded on a Mono-S analytical column. Elution was performed with a 20CV gradient of 0–15% using 20 mM sodium phosphate buffer pH = 6.2 and 1 M NaCl. For IEX-MALS experiments, solvent parameters were corrected based on the conductivity of 85 mM NaCl: viscosity at 25 °C was defined as 0.8974 cP and refractive index at 658.9 nm was defined as 1.3317.

A 16-residues peptide (sequence: *WTEEFVEKMLEDLEDL*) with a theoretical molar mass of 2026 Da was synthesized on a Liberty microwave-assisted peptide synthesizer (CEM) using standard Fmoc chemistry on rink amide resin. The peptide was cleaved from the resin by 3 hours shaking in a mixture of 95% TFA, 2.5% TDW and 2.5% TIPS and purified on a Merck-Hitachi HPLC using a reverse-phase C8 column. MALDI-TOF mass spectrometry and analytical HPLC were used to verify the identity and purity of the peptide. 2 mg peptide was dissolved in 20 mM Tris-HCl buffer pH = 8.5 and 9 mL of 0.22 mg/mL were loaded and analyzed with AIEX-MALS using a Mono-Q analytical column. Elution was performed with a 35% step of 20 mM Tris-HCl buffer pH = 8.5 and 1 M NaCl.

### Native and SDS-PAGE protein gels

Commercial BSA, fibronectin and ovalbumin were analyzed with 4–15% SDS-PAGE gels (Bio-Rad 4568086) in reducing conditions. Peaks from Mono-Q runs of BSA and ovalbumin were also analyzed with these 4–15% SDS-PAGE gels with same conditions. Different fractions from the Mono-S run of Adalimumab were analyzed with 8–16% SDS-PAGE gel (GenScript M81615) in reducing and non-reducing conditions. Gels were stained by an Instant Blue Coomassie staining solution (Expedeon) and exposed with ChemiDoc XRS + camera (Bio-Rad).

Several fractions from the Mono-Q run of hoefavidin variant were analyzed using native PAGE^32^. 15 µl of the sample was incubated with 1 µl of 0.4 mM Biotin-4- Fluorescein for 30 min and then separated and analyzed on a native acryl amide gel. Gel was exposed with ChemiDoc XRS + camera (Bio-Rad).

## Electronic supplementary material

Supplementary Information
