# Publisher Correction: Coupling Multi Angle Light Scattering to Ion Exchange chromatography (IEX-MALS) for protein characterization

**DOI:** 10.1038/s41598-019-41391-y

**Published:** 2019-04-11

**Authors:** Hadar Amartely, Orly Avraham, Assaf Friedler, Oded Livnah, Mario Lebendiker

**Affiliations:** 1 Wolfson Centre for Applied Structural Biology, The Alexander Silberman Institute of Life Sciences, The Hebrew University of Jerusalem, Safra Campus, Givat Ram, Jerusalem, Israel; 2 Department of Biological Chemistry, The Alexander Silberman Institute of Life Sciences, The Hebrew University of Jerusalem, Safra Campus, Givat Ram, Jerusalem, Israel; 3 Institute of Chemistry, The Hebrew University of Jerusalem, Safra Campus, Givat Ram, Jerusalem, Israel

Correction to: *Scientific Reports* 10.1038/s41598-018-25246-6, published online 02 May 2018

This Article contains in-text referencing errors.

Table 1 contains an error in the legend where,

“*Injected volume is unlimited, although total amount of injected sample is restricted by the column capacity. For Mono Q HR 5/5 (GE) the loading capacity is ~25 mg protein^33^.”

should read:

“*Injected volume is unlimited, although total amount of injected sample is restricted by the column capacity. For Mono Q HR 5/5 (GE) the loading capacity is ~25 mg protein^29^.”

In the Materials and Methods section, under the subheading ‘IEX-MALS experiments’,

“Equation 1^29^ was used for the correction of dn/dc value for each eluted peak in IEX-MALS experiments:”

should read:

“Equation 1^30^ was used for the correction of dn/dc value for each eluted peak in IEX-MALS experiments:”

In the same section,

“The specific protein volume - $\bar{v}$ was determined as 0.73 mL/g, an average value for proteins^30^. Increase of 0.00085 in n (solvent), equals to increase of 85 mM NaCl, leading to decrease of 0.0011 in dn/dc^31^.”

should read:

“The specific protein volume - $\bar{v}$ was determined as 0.73 mL/g, an average value for proteins^31^. Increase of 0.00085 in n (solvent), equals to increase of 85 mM NaCl, leading to decrease of 0.0011 in dn/dc^32^.”

Furthermore,

“For every 85 mM shift in NaCl concentration, a change of 0.008 cP in the viscosity is introduced^31^.”

should read:

“For every 85 mM shift in NaCl concentration, a change of 0.008 cP in the viscosity is introduced^32^.”

Finally, in the Materials and Methods section, under the subheading ‘Native and SDS-PAGE protein gels’,

“Several fractions from the Mono-Q run of hoefavidin variant were analyzed using native PAGE^32^.”

should read:

“Several fractions from the Mono-Q run of hoefavidin variant were analyzed using native PAGE^33^.”

