# Supplementary material for: Coupling Multi Angle Light Scattering to Ion Exchange chromatography (IEX-MALS) for protein characterization
Source: Sci Rep. 2018 May 2;8:6907. doi: 10.1038/s41598-018-25246-6 (PMC5931992; doi:10.1038/s41598-018-25246-6)
Supplement: Supplementary file 1 — Supplementary Information [file 41598_2018_25246_MOESM1_ESM.pdf]

Supplementary information – Coupling Multi Angle Light Scattering to Ion Exchange chromatography (IEX-MALS) for protein characterization

Hadar Amartely, Orly Avraham, Assaf Friedler, Oded Livnah and Mario Lebendiker

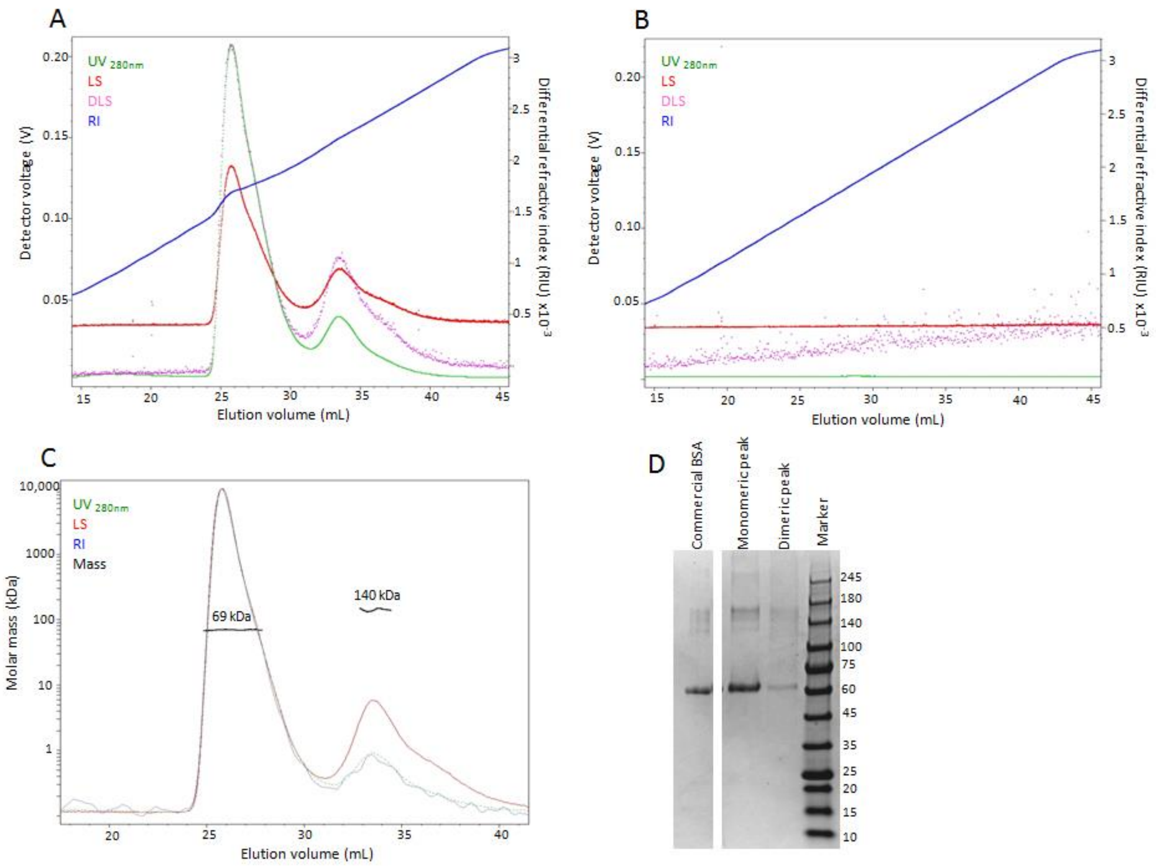

Figure S1: AIEX-MALS analysis of BSA including RI baseline subtraction. BSA was separated using a monoQ HR 5/5 column and analyzed using an in-line MALS instrument. A) A basic collection chromatogram of BSA separation on AIEX with a 15-70% gradient. B) A basic collection chromatogram of the same gradient program without protein injection. C) BSA elution profile with mass analysis achieved by MALS after RI buffer subtraction. The chromatograms display the UV at 280nm (green), light scattering at 90° angle (red), refractive index (blue) and dynamic light scattering (pink) curves. Molar masses of the peaks (black) were calculated using RI as the concentration source for MALS calculations. D) Gel image of SDS-PAGE with Coomassie staining analysis of BSA before and after separation in AIEX (see Supplementary fig. S4 for full gel image).

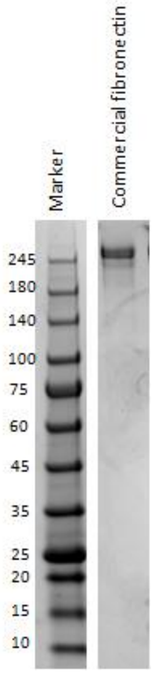

Figure S2: Purity of fibronectin. Gel image of SDS-PAGE with Coomassie staining analysis of the commercial fibronectin. Full gel image displays in Supplementary fig. S4.

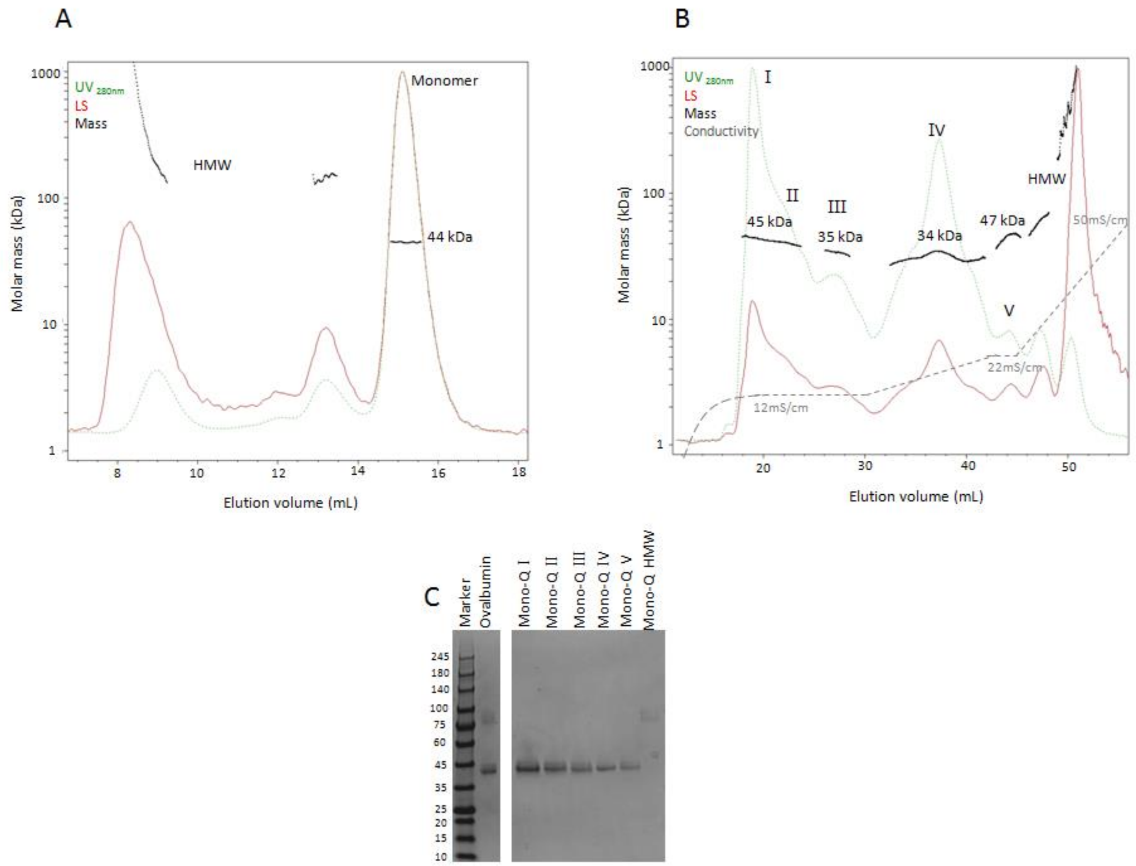

Figure S3: SEC-MALS and AIEX-MALS analysis of ovalbumin. Ovalbumin was separated and analyzed using an analytical SEC column Superdex 200 increase (A) and an AIEX analytical column Mono-Q consequently with MALS. The chromatograms display the UV at 280nm (green), light scattering at 90° angle (red), refractive index (blue) and conductivity (grey) curves together with the molar mass of each peak calculated by MALS (black). C) Gel image of SDS-PAGE with Coomassie staining analysis of ovalbumin before and after separation in AIEX (see Supplementary fig. S4 for full gel image).

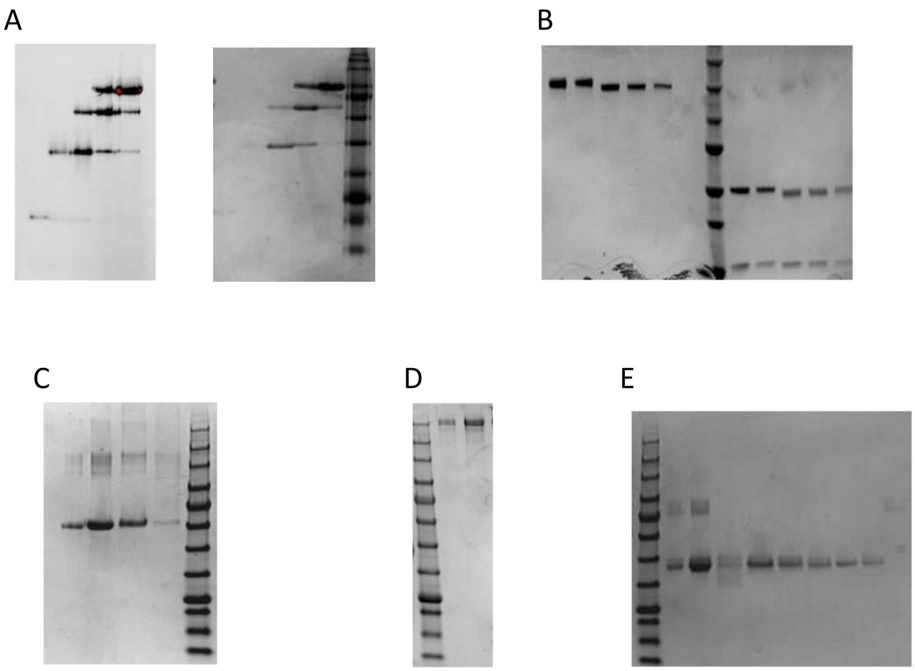

Figure S4: Original gel images for figure 4 (A) and figure 5 (B) of the main manuscript and for figure S1 (C), figure S2 (D) and figure S3 (E) of the supplementary information.
